# mtROS-NF-κB signaling supports ILC3 function and survival during sepsis-induced intestinal injury

**DOI:** 10.3389/fimmu.2026.1843175

**Published:** 2026-09-09

**Authors:** Limin Chen, Haochen Zhang, Sisi Huang, Yizhu Chen, Dongdi Wang, Xiaojun Pan, Lidi Zhang, Zhenliang Wen, Xiaohui Su, Sheng Zhang, Jiao Liu, Lei Shen, Dechang Chen

**Affiliations:** 1 Department of Critical Care Medicine, Ruijin Hospital, Shanghai Jiao Tong University School of Medicine, Shanghai, China; 2 Center for Immune-Related Diseases at Shanghai Institute of Immunology, Ruijin Hospital, Shanghai Jiao Tong University School of Medicine, Shanghai, China; 3 Department of Immunology and Microbiology, Key Laboratory of Cell Differentiation and Apoptosis of Chinese Ministry of Education, Shanghai Jiao Tong University School of Medicine, Shanghai, China

**Keywords:** colonic injury, ILC3s, mitochondrial impairment, mtROS, NF-κB, sepsis

## Abstract

Sepsis frequently induces intestinal barrier injury, which exacerbates systemic inflammation, and contributes to high mortality. Group 3 innate lymphoid cells (ILC3s) are key regulators of mucosal immunity, yet their role in sepsis-associated intestinal injury remains incompletely understood. We found circulating ILCPs were reduced in patients with sepsis. In an LPS-induced murine sepsis model, colonic ILC3 numbers declined whereas the residual population showed increased frequencies of IL-22- and GM-CSF-producing cells. RORγt-deficient mice exhibited impaired induction of colonic IL-22 and GM-CSF, increased intestinal permeability, and aggravated histopathological injury. Adoptive transfer of purified wild-type ILC3s restored colonic IL-22 and GM-CSF levels and partially improved barrier integrity. Mechanistically, septic ILC3s showed hypoxia-associated mitochondrial impairment and accumulation of mitochondrial ROS. increased mtROS contributed to enhanced NF-κB p65 phosphorylation and selectively supported IL-22 and GM-CSF production. Recombinant IL-22 or GM-CSF ameliorated intestinal injury. However, ROS scavenging or NF-κB inhibition increased ILC3 apoptosis, indicating that ROS-NF-κB axis also supports residual ILC3 survival. Together, these findings identify a mitochondria-associated ROS-NF-κB program that sustains protective cytokine production and survival in residual intestinal ILC3s during sepsis, although it is insufficient to prevent ongoing ILC3 loss and intestinal injury.

## 1 Introduction

Sepsis, a life-threatening organ dysfunction caused by a dysregulated host response to infection (1), remains a leading cause of mortality in intensive care units (ICUs) worldwide (2). Among the multiple organ systems affected, intestinal barrier injury is increasingly recognized as a critical event in septic patients (3). The disruption of gut barrier integrity during sepsis, which permits the translocation of luminal bacteria, metabolites, and pathogen-associated molecular patterns such as lipopolysaccharide (LPS) into the systemic circulation, plays a pivotal role in driving systemic inflammation and the development of multiple organ failure (4, 5). Therefore, targeting and interrupting this detrimental cycle between intestinal barrier disruption and systemic inflammation represents a promising therapeutic strategy for sepsis.

Innate lymphoid cells (ILCs) are a family of lymphocytes that lack antigen-specific receptors and function as key sentinels and regulators at mucosal barriers (6). Among them, group 3 innate lymphoid cells (ILC3s), characterized by their expression of the transcription factor retinoid acid-related orphan receptor gamma t (RORγt), constitute the predominant ILC subset in the intestinal lamina propria (7). ILC3s are crucial for maintaining intestinal barrier integrity primarily through the secretion of cytokines such as interleukin (IL)-22, IL-17, and granulocyte-macrophage colony-stimulating factor (GM-CSF). These cytokines promote epithelial cell proliferation, stimulate intestinal stem cell differentiation, induce antimicrobial peptide (AMP) production from epithelial cells, and aid in coordinating adaptive immune responses, thereby positioning ILC3s at the center of mucosal anti-infective defense (8–10). However, accumulating evidence from intestinal inflammation models suggests that ILC3s may exhibit a “double-edged sword” role. Dysregulated ILC3s may contribute to immunopathology through excessive or altered production of cytokines such as IL-17, IL-22, and interferon (IFN)-γ, contributing to chronic inflammatory disorders including inflammatory bowel disease (11, 12). Despite their well-established importance in intestinal immunity, the specific role, functional dynamics, and regulatory mechanisms of ILC3s during sepsis-induced intestinal injury remain poorly understood.

Accumulating evidence indicates that the activation and subsequent cytokine production of ILC3s are regulated by oxygen levels, linking their function to hypoxic signaling and metabolic adaptation. In particular, mitochondrial reactive oxygen species (mtROS) have been shown to stabilize hypoxia-induced factor (HIF)-1α and RORγt, thereby integrating glycolytic metabolism with transcriptional regulation during ILC3 activation (13). Recent researches further demonstrate that hypoxic environment regulates intestinal ILC3 proliferation, activation, and cytokine production via the HIF-1 signaling pathway (14, 15). Ischemia and tissue hypoxia are hallmark histopathological features of sepsis, where inflammatory factors and oxidative stress lead to increased cellular production of reactive oxygen species (ROS) (16, 17). Beyond their classical role in oxidative damage, ROS function as critical signaling intermediates regulating cell metabolism, differentiation, and survival (18–20). However, whether oxidative stress regulates ILC3s during sepsis, and the underlying signaling pathways involved, remain entirely unexplored.

Our study reveals that during sepsis-induced intestinal injury, the number of ILC3s decreased, while paradoxically, their IL-22 and GM-CSF producing capacity was enhanced. Nevertheless, the ILC3 derived cytokines IL-22 and GM-CSF exerted protective effects and repair of intestinal barrier damage. Here, ROS promotes nuclear factor κ-light-chain-enhancer of activated B cells (NF-κB) p65 phosphorylation level and nuclear translocation to promote residual ILC3 survival and production of IL-22 and GM-CSF in sepsis mice colon.

## 2 Materials and methods

### 2.1 Human sample collection

Human peripheral blood samples were obtained from patients with sepsis or healthy donors. Patients aged between 18 and 80 years who were admitted to the ICUs with a confirmed diagnosis of sepsis were included in the study and those receiving immunosuppressive therapy or with immunodeficiency conditions were excluded. Samples were obtained from the Department of Critical Care Medicine, Ruijin Hospital Affiliated to Shanghai Jiao Tong University School of Medicine. All participants provided informed consent and this study was approved by the Ethical Committee of the Ruijin Hospital Affiliated to Shanghai Jiao Tong University School of Medicine (Approval No.2021-213). PBMCs and plasma were isolated from whole blood with Ficoll-Paque PLUS (GE Healthcare) according to the manufacturer’s guidelines.

### 2.2 Mice

Wild-type mice (C57BL/6) were purchased from Shanghai Laboratory Animal Center. *Rorc*^gfp/gfp^ mice (Strain:007572) on C57BL/6 background were purchased from the Jackson Laboratory. All mice were bred and maintained at Phenotek Biotechnology (Shanghai, China, certification NO.SYXK (SH) 2018-0023) Co., Ltd in SPF facilities with a 12 h/12 h light-dark cycle, an average ambient temperature of 25°C, an average humidity of 50%, and were given the same and regular diet. 8-10-week-old male mice weighing 20 to 25g were used in this study. All animals were euthanized by intraperitoneal injection of an overdose of sodium pentobarbital (150 mg/kg). All experiments were approved by the Committee for Animal Policy and Welfare of Ruijin Hospital Affiliated to Shanghai Jiao Tong University School of Medicine and performed in accordance with the institutional and national guidelines.

### 2.3 Mouse models of sepsis

Mice were injected intraperitoneally with lethal doses (20 mg/kg body weight) of LPS derived from Escherichia coli O111:B4 (Sigma-Aldrich) diluted with PBS and PBS as control. Tissues were harvested 0, 12, 24 hours post-injection.

### 2.4 Cytokine supplementation and neutralization in mice

Mice were randomly assigned to the indicated treatment groups. For cytokine supplementation, recombinant mouse GM-CSF (BioLegend) and IL-22 (R&D) were administered intraperitoneally at a dose of 500ng per mouse 4 h prior to LPS challenge. For cytokine neutralization, mice were treated intraperitoneally with anti-GM-CSF (BioLegend) or anti-IL-22 (BioLegend) neutralizing antibodies at a dose of 50 μg per mouse 4 h before and 6 h after LPS challenge. Control mice received isotype control antibodies. Serum and intestinal tissue were harvested 12 h after LPS challenge for downstream analyses.

### 2.5 Adoptive transfer of ILC3s

To determine whether ILC3s are sufficient to protect intestinal barrier integrity during sepsis, and to exclude potential contributions from other RORγt^+^cell populations (e.g., Th17 cells, RORγt^+^ antigen-presenting cells), 2×10^5^ ILC3s were sorted from WT mice, and adoptively transferred intravenously into *Rorc*^gfp/gfp^ mice (KO) mice. Age-matched Rorc*^gfp/gfp^* littermates without adoptive transfer were used as controls. Four days after engraftment, sepsis was induced by intraperitoneal LPS injection, and intestinal barrier function was evaluated at 12 hours by measuring intestinal permeability and histological scoring of intestinal tissue sections.

### 2.6 Intestinal permeability assay

Intestinal barrier permeability was assessed using FITC-dextran (40 kDa; Chondrex). Mice were fasted for 8 hours prior to oral gavage with FITC-dextran. Four hours after gavage, blood was collected via retro-orbital bleeding into heparinized tubes. Plasma was separated by centrifugation. Fluorescence intensity was measured using a microplate reader at excitation/emission wavelengths of 485/535 nm, and FITC-dextran concentrations were calculated against a standard curve of serially diluted FITC-dextran in control plasma.

### 2.7 Histologic analysis

Segments of large intestine from mice were immediately fixed in 4% paraformaldehyde. The tissues were then embedded in paraffin and cut transversely into 7µm sections. Sections were stained with hematoxylin and eosin (H&E) or Alcian Blue–Periodic Acid–Schiff (AB-PAS) to assess mucin production and were examined by light microscopy. Crypt depth was measured in well-oriented colonic crypts on H&E-stained sections using CaseViewer software. The distance from the crypt base to the luminal surface was measured in at least 5 intact crypts per mouse, and the mean value for each mouse was used for statistical analysis.

### 2.8 Isolation of lamina propria immune cells from the intestines of mice

Large intestines were removed from mice and cleaned by removing fat or scar tissues. The intestines were opened longitudinally and extensively cleaned with PBS, then cut into 0.5 cm long pieces. PBS containing 1 mM DTT and 30 mM EDTA was used to remove epithelial cells by shaking for 10 min, followed by an additional 10 min shaking in PBS with 30 mM EDTA to remove residual DTT. The intestinal tissue was digested with RPMI 1640 medium containing 10% FBS (Gibco), collagenase VIII (200 U/mL) and DNase I (150 μg/mL) at 37°C for 90 min. Mononuclear cells were further enriched by a 40%-80% Percoll density gradient centrifugation at room temperature for 20 min.

### 2.9 Flow cytometry and cell sorting

Dead cells were identified with LIVE/DEAD™ Fixable Near-IR Dead Cell Stain Kit (Thermo Fisher).

For human samples, Fc receptors were blocked with Human TruStain FcX™ (Bioglengd) for 5 min at room temperature. Single-cell suspensions were then stained with antibody cocktail in FACS buffer (2% FBS and 0.5mM EDTA in PBS) at 4 °C for 30 min. The following surface antibodies were used at a 1:200 dilution: CD1a, CD3, CD19, CD20, CD14, CD16, CD34, CD45, CD94, CD117, CD123, CD127, CD161, CD303, TCRαβ, TCRγδ, FcϵR1a, CRTH2, NKp44. Human lineage marker included CD1a, CD3, CD19, CD20, CD14, CD16, CD34, CD94, CD123, CD303, TCRαβ, TCRγδ. Circulating helper-like ILCs were defined as lineage-negative (Lin^-^) CD127^+^cells. Within the Lin^-^CD127^+^ population, ILC2s were identified as CRTH2^+^cells, ILC1-like cells as CRTH2^-^CD117^-^cells, and ILC precursors (ILCPs) as CRTH2^-^CD117^+^cells which may include both mature ILC3s and ILC precursors (21–23).

For mouse samples, Fc receptors were blocked with anti-CD16/CD32 (Bioglengd) for 5 min at room temperature. Single-cell suspensions were stained with antibody cocktail in FACS buffer (2% FBS and 0.5mM EDTA in PBS) at 4 °C for 30 min. For transcription factor staining and Ki67 proliferation assays, cells were fixed and permeabilized using the Foxp3/TF staining buffer sets (eBioscience), followed by staining in 1× permeabilized buffer containing antibody at 4 °C for 45 min. For cytokines staining, cells were pre-incubated for 4 hours with ionomycin (500 ng/mL), phorbol myristate acetate (50 ng/mL), and brefeldin A (2 μg/mL), and then fixed with Foxp3/TF staining buffer sets (eBioscience) before staining.

For cell sorting, dead cells were identified with DAPI (Biolegend). Intestinal ILC3s were further sorted as Lin-(CD3ϵ, CD11b, CD11c, CD45R, GR-1)CD45^mid^CD90^+^KLRG-1^-^NK1.1^-^ live cell.

The following surface antibodies were used at a 1:200 dilution: CD3ϵ, CD11b, CD11c, CD45, CD45R, CD90, GR-1, CCR6, NKp46, NK1.1, KLRG-1. The following antibodies were used at a 1:100 dilution: RORγt, T-bet, Gata3, Ki67, IL-22, IL-17A, IFN-γ, GM-CSF.

Conjugated antibodies were purchased from Biolegend, eBioscience, or BD Biosciences. All flow cytometry experiments were performed on Fortessa flow cytometers (BD Biosciences) and analyzed with FlowJo software. A FACS Aria III cell sorter (BD Biosciences) was used for cell sorting.

### 2.10 Multiplex cytokine analysis

Plasma or tissue samples were collected and stored at -80 °C until analysis. For tissue cytokine measurements, intestinal tissues were homogenized in PBS containing protease inhibitors and supernatants were collected after centrifugation. Cytokine concentrations were measured using LEGENDplex™ multiplex cytokine assay (BioLegend) according to the manufacturer’s protocol. Cytokine concentrations were calculated based on standard curves generated using recombinant cytokine standards and expressed as pg/mL or normalized to tissue weight or total protein concentration.

### 2.11 Cellular metabolic assay

For mitochondrial mass, membrane potential and glucose uptake assay, cells were incubated with MitoTracker™ Green FM (Thermo Fisher Scientific), MitoTracker™ Deep Red FM (Thermo Fisher Scientific), TMRE (Thermo Fisher Scientific), or 2-NBDG (Thermo Fisher Scientific) at 37 °C for 30 min before surface staining as described above.

For cellular and mitochondria ROS assay, cells were incubated with H2BCFDA (Thermo Fisher Scientific) or MitoSOX (Thermo Fisher Scientific) at 37 °C for 30 min before surface staining as described above.

### 2.12 Cellular adenosine triphosphate (ATP) detection

Cellular ATP levels were measured using an ATP Assay Kit (Beyotime) according to the manufacturer’s instructions. Sorted intestinal ILC3s were collected and lysed using the lysis buffer provided in the kit. ATP concentrations were calculated based on a standard curve generated with ATP standards provided in the kit and normalized to cell number.

### 2.13 Apoptosis assay

For Annexin V apoptosis assay, after surface markers staining, cells were stained with Annexin V from Annexin V Apoptosis Detection Kit (Biolegend). DAPI (Biolegend) or 7-AAD (Biolegend) was used as viability staining.

For caspase-3 apoptosis assay, after surface markers staining, cells were fixed and permeabilized with BD Cytofix/Cytoperm kit (BD Biosciences) at 4°C for 45 min, then stained with caspase-3 antibody for 45min. For cleaved-caspase-3 (CST), after surface markers staining, cells were fixed and permeabilized with the Foxp3/TF staining buffer sets (eBioscience), then stained with cleaved-caspase-3 antibody for 45min followed by secondary flow fluorescent antibody (Abcam).

### 2.14 RNA extraction and qPCR

Total RNA was extracted from tissue or sorted cell using TRIzol reagent (Invitrogen). cDNA was transcribed with PrimeScript RT Master Kit (Takara). Real-time quantitative PCR was performed using TB Green Premix Ex Taq II (Tli RNase H Plus) (Takara), and reactions were run with the QuantStudio 7 (Thermo Fisher Scientific). The results are displayed as relative expression values normalized to *Actb*.

### 2.15 Immunofluorescent microscopy

Sorted intestinal ILC3s were first fixed and permeabilized using the Foxp3/TF staining buffer sets (eBioscience) for 45 min at 4 °C. Cells were stained with phospho-NF-κB p65 (Ser536) (CST) for 30 min at room temperature, followed by secondar antibody staining for 30 min at room temperature. Then cells were transferred to adhesion microscope slides (Citotest) using CYTOSPIN 4 (Thermo Scientific). The slides were mounted with DAPI Fluoromount-G™ (Yeasen) and analyzed by confocal microscopy (LEICA DM6B).

### 2.16 Immunoblot analysis

Sorted large intestinal ILC3 cells were lysed in protein lysis buffer (50mM Tris-HCl, 2% SDS, and 10% Glycerol in deionized water) containing protein inhibitor (Thermo Fisher Scientific) and phosphatase inhibitor (Thermo Fisher Scientific) on ice. Protein concentrations were determined by BCA Protein Concentration Assay (Epizyme). The supernatants were mixed with SDS-PAGE loading buffer (Beyotime) and incubated at 100 °C for 5 min. Proteins were separated on 12% Tris-Glycine gels and transferred onto Immobilon^®^-P PVDF Membrane (Merck). The membrane was blocked in 5% non-fat dried milk for 2 hours and incubated overnight with the primary antibodies at 4 °C. The corresponding HRP-conjugated secondary antibodies were added to the membranes for 2 hours at room temperature. Detections were performed with ECL western blotting detection reagents (Millipore) using Amersham Imager 600.

### 2.17 RNA-seq

Intestinal ILC3s were sorted from mice challenged with LPS (20mg/kg) or PBS control. Total RNA was extracted from the sorted intestinal ILC3s using TRIzol reagent. RNA quality was assessed with an Agilent Bioanalyzer 2100. High-quality full-length cDNA was synthesized with the SMARTer™ PCR cDNA Synthesis Kit (Takara Bio USA). Sequencing libraries were generated using the TruePrep DNA Library Prep Kit V2 for Illumina, following the manufacturer’s protocol. The libraries were sequenced on the Illumina Novaseq™ 6000 sequence platform. Raw reads were aligned to the mouse reference genome (version mm10) using HISAT2 RNA-seq alignment software. Relative mRNA expression levels (FPKM) were quantified based on exon regions using Cufflinks and the mm10 genome annotation. Protein-coding genes were used for Gene Set Enrichment Analysis (Broad Institute). Differentially expressed genes identified by DESeq2 (fold change >1.5, *p* < 0.05) were used for Gene Ontology (GO) analysis. Heat maps of the normalized gene counts were generated using Morpheus. RNA-sequencing data have been deposited in GEO under the primary accession code.

### 2.18 Statistical analysis

Statistical analyses were performed using GraphPad Prism 10.1.2. Unless otherwise noted, statistical significance was determined by the two-tailed unpaired Student’s t test for comparing two groups, by one-way or two-way analysis of variance analysis (ANOVA) on individual biological samples. Detailed information on the statistical analyses is included in the figure legends. Data from the experiments are presented as mean values ± SEM. *p*-values are noted in each figure.

## 3 Results

### 3.1 Peripheral circulating ILC2 and ILCPs decrease in sepsis patients

**Figure 1:**
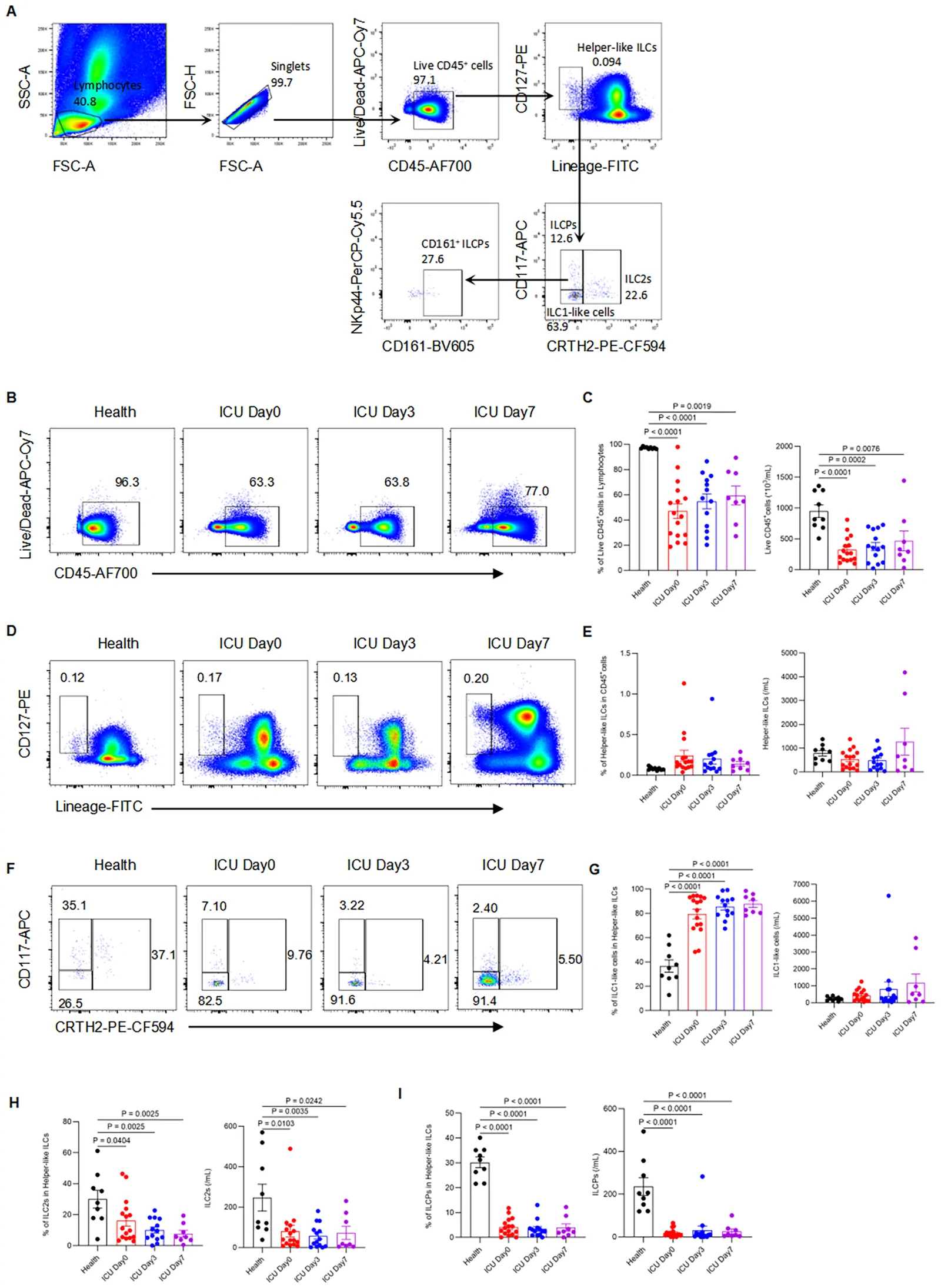
Circulating ILCPs decrease in peripheral blood of septic patients. **(A)** Gating strategy of human helper like ILCs and subsets in peripheral blood. Circulating helper-like ILCs were defined as lineage-negative (Lin^-^)CD127^+^cells. Within the Lin^-^CD127^+^ population, ILC2s were identified as CRTH2^+^cells, ILC1-like cells as CRTH2^-^CD117^-^cells. Circulating CD117^+^CRTH2^-^ ILCs were identified as ILCPs. Representative flow cytometry plots **(B)** and frequency **(C)** of CD45^+^cells within total live lymphocytes in peripheral blood of HCs (n = 9) and septic patients on ICU 0 day (n = 16), 3 days (n = 13), and 7 days (n = 8). Representative flow cytometry plots **(D)** for ILCs gated on Lin^-^CD45^+^ and **(E)** frequency of total ILCs within CD45^+^cells in peripheral blood of health control (HCs) (n = 9) and septic patients on ICU 0 day (n = 16), 3 days (n = 13), and 7 days (n = 8). Representative flow cytometry plots **(F)** for ILC1-like cells (CD117^-^CRTH2^-^), ILC2s (CD117^-^CRTH2^+^), and ILCPs (CD117^+^CRTH2^-^) gated on Lin^-^CD45^+^CD127^+^. Frequency and absolute number of ILC1-like cells **(G)**,ILC2 **(H)** and ILCPs **(I)** in peripheral blood of HCs (n = 9) and septic patients on ICU 0 day (n = 16), 3 days (n = 13), and 7 days (n = 8). Data are presented as mean ± SEM. Each symbol represents an individual sample. For statistical analysis, the following tests were used. One-way ANOVA followed by Tukey’s multiple comparisons test in **(C, E, G, H, I)**. Each symbol represents an individual sample. *p*-values are shown on the graphs, and *p*-values < 0.0001 are reported as such.

To characterize alterations in the circulating helper-like ILC compartment during septic patients, we analyzed peripheral blood ILC populations in healthy controls and patients with sepsis at ICU admission (day 0), as well as on day 3 and day 7 of ICU stay. No significant difference was observed in Sequential Organ Failure Assessment (SOFA) scores or Acute Physiology and Chronic Health Evaluation (APACHE) II score among patients on these timepoints (**Supplementary Figures 1A, B**). Flow cytometry was used to analyze changes in ILC populations in peripheral blood lymphocytes. The gating strategy of human circulating ILC populations is shown in **Figure 1A** as previously described (21, 22, 24). We found that, in patients with sepsis, the frequency and absolute number of live CD45^+^ cell within peripheral lymphocytes decreased (**Figures 1B, C**), while the proportion and number of total helper-like ILCs among CD45^+^ cells showed no significant change (**Figures 1D, E**).

Notably, analysis of ILC subsets showed that the proportion of circulating ILC1-like cells increased markedly while the number of ILC1-like cells remained unchanged (**Figures 1F, G**), whereas both the proportion and absolute number of ILC2 (**Figures 1F, H**) and circulating ILCPs (**Figures 1F, I**) were significantly reduced. Although CD161 and NKp44 were included in the flow-cytometry panel, the number of circulating ILCPs events in patients with sepsis was insufficient for reliable downstream subdivision and statistical comparison according to CD161 or NKp44 expression. These subpopulation analyses were therefore not performed. Together, these data establish that sepsis is associated with a preferential and disproportionate reduction of ILC2 and circulating ILCPs.

To determine whether the reduction in ILC2s and ILCPs was specifically associated with sepsis secondary to intestinal perforation, we further stratified septic patients into two subgroups: those with intestinal perforation and those without. No differences were found in SOFA scores (**Supplementary Figure 1C**) between the two groups. Neither the absolute counts of the white blood cells, neutrophils, lymphocytes, or monocytes (**Supplementary Figure 1D**), nor the number or frequency of CD45^+^ cells (**Supplementary Figure 1E**), helper-like ILCs (**Supplementary Figure 1F**), the ILC1-like cells (**Supplementary Figure 1G**), the ILC2s (**Supplementary Figure 1H**) or ILCPs (**Supplementary Figure 1I**) had any change. These data suggest that the depletion of circulating ILC2s and ILCPs may be a generalized response to sepsis rather than specifically linked to intestinal perforation.

### 3.2 Dynamics and functional activation of ILC3s in mice colon during sepsis

ILC3s are primarily tissue-resident cells that exert their functions at intestinal mucosal barriers (25, 26). Given that circulating ILCPs cannot be equated with mature ILC3s and may not reflect tissue-resident ILC responses, and direct sampling of intestinal tissue from septic patients is ethically and practically unfeasible, we employed a complementary murine sepsis model induced by LPS.

Mice were challenged by intraperitoneal injection with 20mg/kg LPS. Compared with PBS-treated controls, LPS-challenged mice began to die between 12- and 24-hours post-injection, with a sharp increase in mortality observed at 36–48 hours (**Supplementary Figure 2A**). At 12 hours, serum levels of pro-inflammatory cytokines including IFN-γ, tumor necrosis factor (TNF)-α, monocyte chemotactic protein (MCP)-1, IL-1β, IL-6, and the anti-inflammatory cytokine IL-10 were significantly elevated (**Supplementary Figure 2B**), confirming the establishment of a hyperinflammatory state. However, although colonic inflammatory cytokines were elevated at 12 hours, they returned to baseline by 24 hours (**Supplementary Figure 2F**). Despite the apparent resolution of local inflammatory mediators, histopathological analysis revealed progressive disruption of intestinal barrier integrity over time (**Supplementary Figures 2C–E**). Meanwhile, antimicrobial peptide expression remained consistently elevated (**Supplementary Figure 2G**). Collectively, these findings indicate that the septic colon is characterized by progressive barrier injury despite sustained antimicrobial responses.

**Figure 2:**
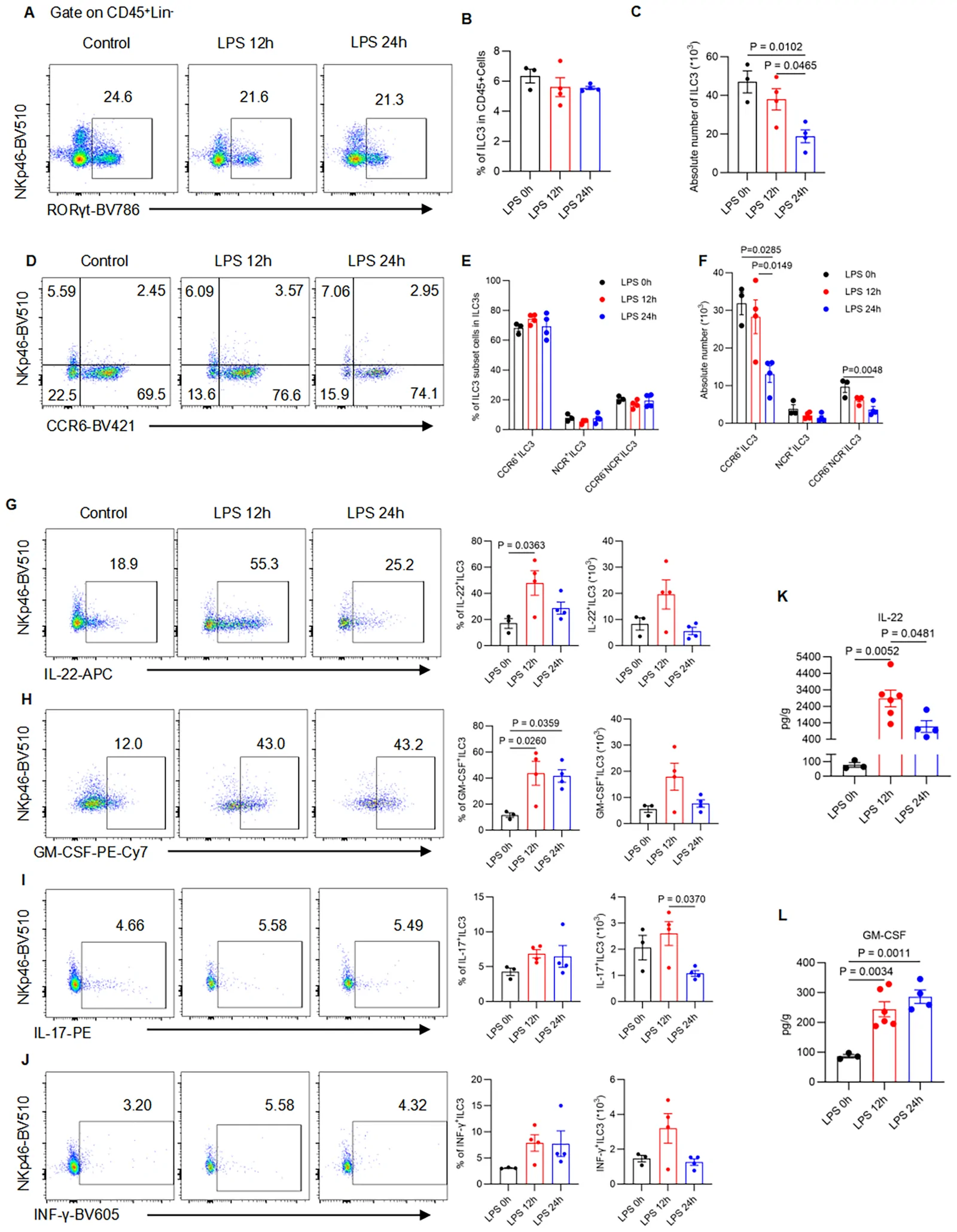
Large intestinal ILC3s are activated during LPS-induced sepsis. Wild type mice were challenged by LPS (20mg/kg) intraperitoneal injection (i.p) to induce sepsis model, while control mice received an equal volume of 1×PBS. Serum and colon tissues were collected and analyzed at 0 h, 12 h and 24h. **(A)** Representative flow cytometry plots for ILC3s gated on Live/dead^-^CD45^+^Lin^-^. Frequency **(B)** and absolute number **(C)** of total ILC3s (RORγt^+^ among Lin^-^CD45^+^) at 0h, 12h, and 24h after LPS i.p (n = 3, 4, 4 from left to right). **(D)** Representative flow cytometry plots for ILC3 subsets gated on Lin^-^CD45^+^RORγt^+^. Frequencies **(E)** and absolute number **(F)** of CCR6^+^ ILC3s, NKp46^+^ ILC3s and NKp46^-^CCR6^-^ ILC3s at 0h, 12h, and 24h after LPS i.p (n = 3, 4, 4 from left to right). **(G)** Representative flow cytometry plots, frequency and absolute number for IL-22 produced by ILC3s gated on Lin^-^CD45^+^RORγt^+^ at 0h, 12h, and 24h after LPS i.p (n = 3, 4, 4 from left to right). **(H)** Representative flow cytometry plots, frequency and absolute number for GM-CSF produced by ILC3s (n = 3, 4, 4 from left to right). **(I)** Representative flow cytometry plots, frequency and absolute number for IL-17 produced by ILC3s (n = 3, 4, 4 from left to right). **(J)** Representative flow cytometry plots, frequency and absolute number for INF-γ produced by ILC3s (n = 3, 4, 4 from left to right). **(K)** The concentration of IL-22 in colon measured by ELISA at 0h, 12h, and 24h after LPS i.p (n = 3, 6, 4 from left to right). **(L)** The concentration of GM-CSF in colon measured by ELISA at 0h, 12h, and 24h after LPS i.p (n = 3, 6, 4 from left to right). Data were pooled from two independent experiments **(A–K)**, shown as mean ± SEM. Statistical significance was determined by one-way ANOVA followed by Tukey’s multiple comparisons test in **(B, C, E, F, G–L)**. Each symbol represents an individual mouse **(B, C, E, F, G–L)**. *p*-values are shown on the graphs, and *p*-values < 0.0001 are reported as such.

In parallel, the absolute number of colonic ILC3s was markedly reduced (**Figures 2A, C**). However, their frequency among CD45^+^ cells remained unchanged (**Figures 2A, B**). No change was observed in the frequency of ILC3 subsets (NKp46^+^, CCR6^+^, and CCR6^−^NKp46^−^) but the number of CCR6^+^ILC3s and CCR6^-^Nkp46^-^ILC3s reduced (**Figures 2D–F**). Although ILC3 numbers declined, the remaining ILC3s exhibited enhanced functional capacity, with significantly increased production of IL-22 and GM-CSF (**Figures 2G, H**). However, the absolute numbers of IL-22^+^and GM-CSF^+^ILC3s did not significantly change (**Figures 2G, H**), suggesting that the increased cytokine-positive fractions compensated for the overall loss of ILC3s and preserved these effector populations. In contrast, the absolute number of IL-17-producing ILC3s decreased without a significant change in its frequency, indicating that the loss of IL-17^+^ILC3s largely paralleled the overall reduction in ILC3 abundance (**Figure 2I**). Neither the frequency nor the absolute number of IFN-γ-producing ILC3s changed significantly. (**Figure 2J**). Consistent with the functional profile of ILC3s, tissue concentrations of IL-22 and GM-CSF increased in parallel with the enhanced cytokine production observed in ILC3s (**Figures 2K, L**). Because cytokine levels were measured in tissue homogenates, these findings indicate an enhanced local cytokine response but do not establish ILC3s as the exclusive cellular source.

Collectively, these findings indicate that progressive intestinal injury was accompanied by numerical loss of ILC3s and selective activation of IL-22- and GM-CSF-producing cells within the residual ILC3 compartment.

### 3.3 ILC3 reconstitution partially restores intestinal barrier protection in RORγt-deficient mice

**Figure 3:**
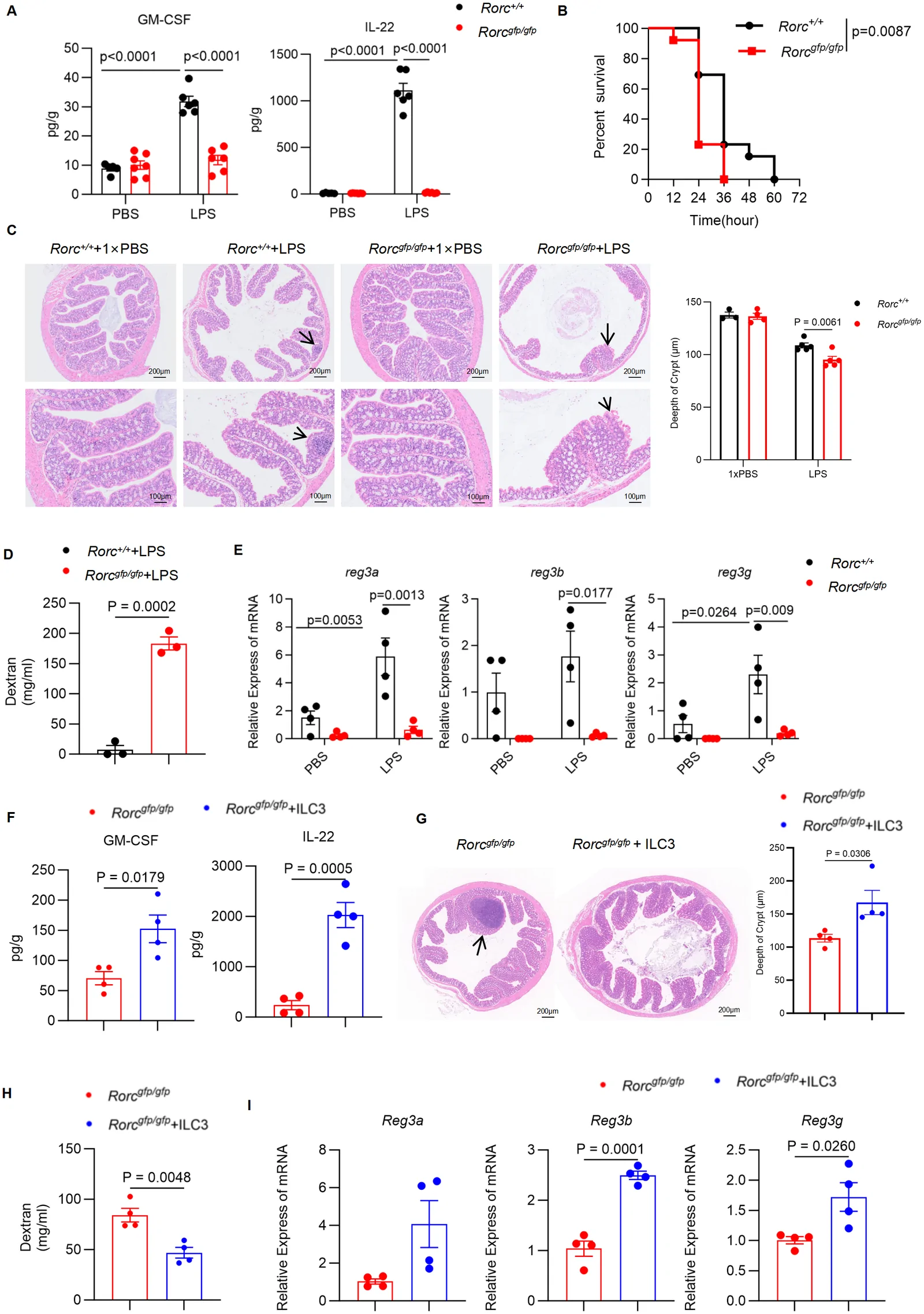
ILC3 reconstitution partially restores intestinal barrier protection in RORγt-deficient mice. *Rorc*^+/+^ and *Rorc*^gfp/gfp^ mice were challenged with 20mg/kg LPS, while control mice received an equal volume of 1×PBS. Colon tissues were collected and analyzed at 12 hours. **(A)** Concentration of GM-CSF and IL-22 in colon from *Rorc*^+/+^ and *Rorc*^gfp/gfp^ mice (n = 5, 7, 6, 6 from left to right). **(B)** Survival curve was monitored every 12 hours for 72 hours after LPS i.p between WT and KO. (n = 13/group, log-rank (Mantel-Cox) test). **(C)** Representative image and crypt depth statistics of H&E staining of colon sections from *Rorc*^+/+^ and *Rorc*^gfp/gfp^ mice in steady state and sepsis. Arrows indicate inflammatory cell infiltration. The scale bar is 200 μm and enlarged to 100 μm. **(D)** Intestinal permeability in *Rorc*^+/+^ and *Rorc*^gfp/gfp^ mice during sepsis detected by dextran assay (n = 4/group). **(E)** Expression of *Reg3a*, *Reg3b*, and *Reg3g* in large intestine from *Rorc*^+/+^ and *Rorc*^gfp/gfp^ mice in steady state and sepsis (n = 4/group). **(F–I)** Sorted ILC3s were transferred to *Rorc*^gfp/gfp^ mice for 4 days. Age-matched Rorc*^gfp/gfp^* littermates without adoptive transfer were used as controls. Sepsis was induced by intraperitoneal LPS injection, and intestinal barrier function was evaluated at 12 hours. **(F)** Concentration of GM-CSF and IL-22 in colon from *Rorc*^gfp/gfp^ and *Rorc*^gfp/gfp^ with ILC3s mice (n = 4/group). **(G)** Representative image of H&E staining of colon sections and statistic of crypt depth from *Rorc*^gfp/gfp^ and *Rorc*^gfp/gfp^ with ILC3s mice. Arrows indicate inflammatory cell infiltration. The scale bar is 200 μm. **(H)** Intestinal permeability in *Rorc*^gfp/gfp^ and *Rorc*^gfp/gfp^ with ILC3s mice during sepsis detected by dextran assay (n = 4/group). **(I)** Expression of *Reg3a*, *Reg3b*, and *Reg3g* in large intestine from *Rorc*^gfp/gfp^ and *Rorc*^gfp/gfp^ with ILC3s mice in sepsis (n = 4/group). Data were pooled from two independent experiments **(A–I)**, shown as mean ± SEM. Statistical significance was determined by log-rank (Mantel-Cox) test **(B)**, two-way ANOVA followed by Tukey’s multiple comparisons test in **(A, C, E)**, two-tailed unpaired Student’s t test in **(D, F–I)**. Each symbol represents an individual mouse **(A, C–I)**. *p*-values are shown on the graphs, and *p*-values < 0.0001 are reported as such.

Given the paradoxical phenotype of ILC3s during sepsis, we next sought to investigate the functional significance of ILC3s in this context. Thus, we utilized RORγt-deficient (*Rorc*^gfp/gfp^) mice. Flow cytometric analysis confirmed efficient depletion of ILC3s in knockout mice (**Supplementary Figure 3A**). Under steady-state conditions, colonic IL-22 and GM-CSF levels were comparable between wild-type (*Rorc*^+/+^) and *Rorc*^gfp/gfp^ mice. Following LPS administration, however, both IL-22 and GM-CSF were markedly increased in the colonic tissue of wild-type mice, whereas this inducible cytokine response was absent or substantially attenuated in *Rorc*^gfp/gfp^ mice (**Figure 3A**). Consequently, colonic IL-22 and GM-CSF levels were significantly lower in LPS-treated *Rorc*^gfp/gfp^ mice than in their wild-type counterparts. Then compared with wild-type controls, *Rorc*^gfp/gfp^ mice exhibited significantly higher mortality following LPS-induced sepsis (**Figure 3B**). No significant differences were observed between WT and *Rorc*^gfp/gfp^ mice in survival or baseline inflammatory cytokine profiles under steady-state (**Supplementary Figure 3B**). During sepsis, *Rorc*^gfp/gfp^ mice demonstrated markedly elevated plasma levels of IFN-γ, IL-1β, and IL-10 relative to septic WT mice (**Supplementary Figure 3B**). Histopathological assessment of the colon revealed no significant structural differences between WT and *Rorc*^gfp/gfp^ mice under steady-state condition (**Figure 3C**). However, following LPS-induced sepsis, *Rorc*^gfp/gfp^ mice developed substantially more severe intestinal injury, characterized by pronounced reduction in crypt depth (**Figure 3C**). Consistent with deterioration of barrier structure, intestinal permeability was significantly increased in septic *Rorc*^gfp/gfp^ mice, assessed by the plasma FITC-dextran concentraion (**Figure 3D**). Under steady-state conditions, *Rorc*^gfp/gfp^ mice exhibited lower baseline AMP expression than WT control (**Figure 3E**). Upon induction of sepsis, WT mice mounted a robust upregulation of AMP, whereas *Rorc*^gfp/gfp^ mice failed to increase AMPs expression, maintaining levels similar to their baseline (**Figure 3E**). These results indicated that RORγt deficiency was associated with impaired intestinal defense during LPS-induced systemic inflammation.

However, because RORγt is expressed across multiple immune-cell populations (27), these findings alone could not attribute the observed protective effect specifically to ILC3. To directly assess the functional contribution of ILC3s, FACS-purified wild-type ILC3s were adoptively transferred into *Rorc*^gfp/gfp^ recipient mice 4 days before LPS administration. Following LPS challenge, ILC3-reconstituted recipients exhibited increased IL-22 and GM-CSF levels compared with non-reconstituted *Rorc*_gfp/gfp_ mice (**Figure 3F**). Importantly, ILC3 adoptive transfer attenuated colonic histopathological injury (**Figure 3G**), reduced serum FITC-dextran concentrations (**Figure 3H**), and increased the expression of intestinal antimicrobial peptide genes (**Figure 3H**).

Collectively, these results indicate that ILC3s contribute directly to the maintenance of intestinal barrier integrity and antimicrobial defense during sepsis. The increase in IL-22 and GM-CSF following ILC3 reconstitution suggests restoration of an ILC3-associated protective response in sepsis-induced colonic injury. We next sought to identify the molecular programs associated with the functional response of residual intestinal ILC3s during sepsis.

### 3.4 NF-κB signaling is enhanced in intestinal ILC3s during systemic inflammation

**Figure 4:**
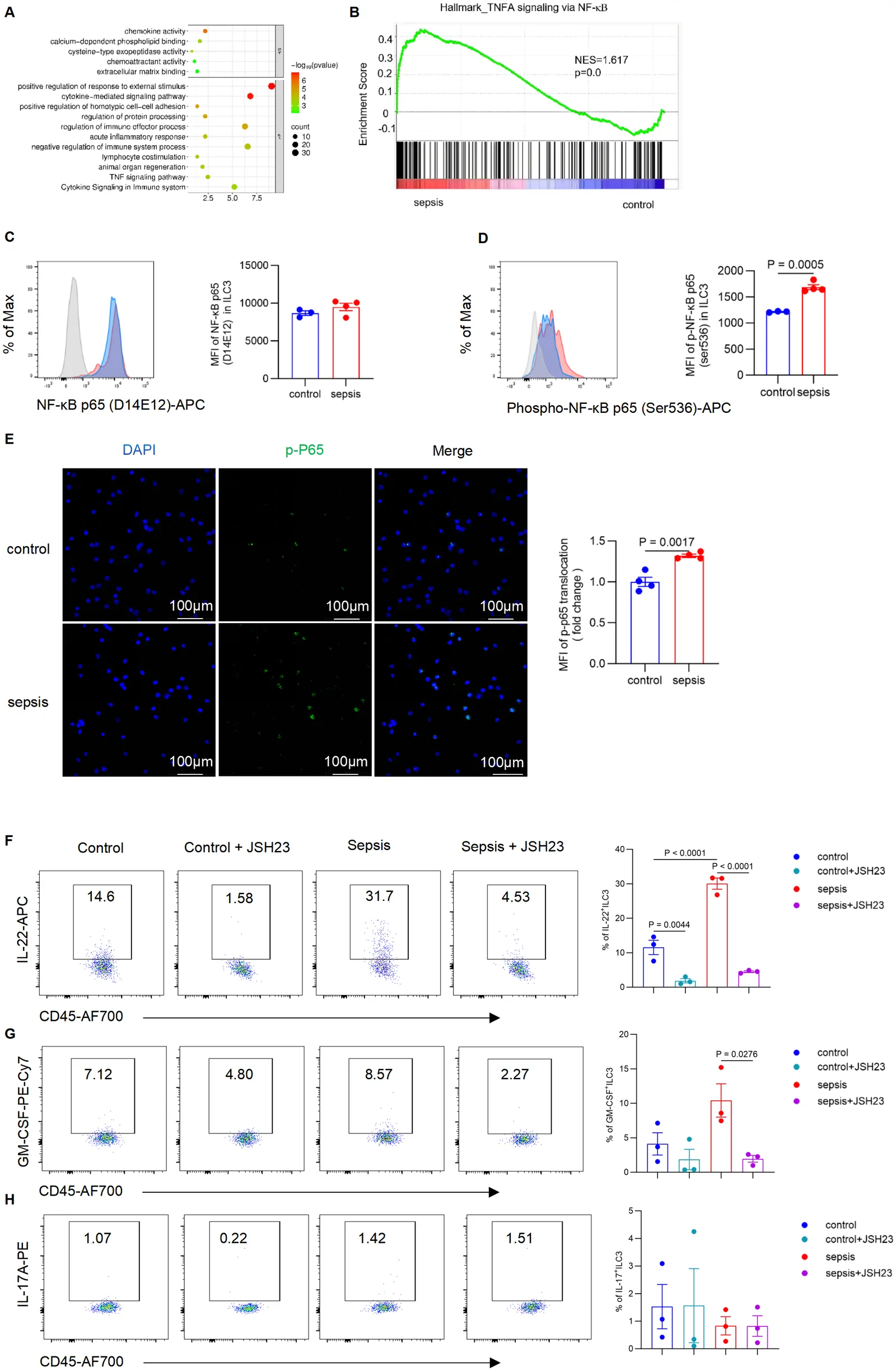
NF-κB signaling increased in colonic ILC3s and regulated the secretion of IL-22 and GM-CSF during sepsis. Wild type mice were challenged with 20 mg/kg LPS i.p, while control mice received an equal volume of 1×PBS. Colon tissues were collected and analyzed at 12 hours. **(A, B)** RNA-seq analysis of sorted ILC3s from PBS- or LPS-treated C57BL/6 wild type mice (n = 3). **(A)** Gene ontology (GO) analysis of pathway upregulated in septic ILC3s. **(B)** Gene set enrichment analysis (GSEA) was performed using the webtool from Broad Institute. NES, normalized enrichment score. Significance was determined by the method implemented in the software (nominal *p* < 0.05). **(C)** The presentive flow cytometry plot and statistic of relative mean fluorescence intensity (MFI) of NF-κB p65 (D14E12) in ILC3s (n = 3,4 from left to right). **(D)** Representative flow cytometry plot and statistic of relative MFI of phosphorylation NF-κB p65 (Ser 536) in ILC3s (n = 3,4 from left to right). **(E)** Immunofluorescence staining of nuclear translocation of p-NF-κB p65 (Green) in colonic ILC3s from control and sepsis mice. Scale bars, 100 μm. And the statistic of relative MFI of p-NF-κB p65 nuclear translocation in colonic ILC3s between control and sepsis mice. **(F–H)** Sorted ILC3s from control and sepsis mice were cultured with JSH-23 (10 μM),the inhibitor of p-NF-κB p65, in the presence of IL-7 (10 ng/ml) for 24 h. Representative flow cytometry plots and frequency of IL-22 **(F)**, GM-CSF **(G)** and IL-17 **(H)** in sorting ILC3 detected after treating with JSH-23,the p-NF-κB p65 inhibitor (n=3/group). Data were pooled from two independent experiments **(A–H)**, shown as mean ± SEM. Statistical significance was determined by a two-tailed unpaired Student’s t test in **(C, D, E)**, one-way ANOVA followed by Tukey’s multiple comparisons test in **(F, G, H)**. Each symbol represents an individual mouse **(C–H)**. *p*-values are shown on the graphs, and *p*-values < 0.0001 are reported as such.

To elucidate the mechanism underlying the functional activation of ILC3s during sepsis, we performed bulk RNA sequencing on colonic ILC3s sorted at baseline (0h) and 12 h post-LPS challenge. Gene Ontology (GO) analysis revealed significant enrichment of pathways associated with the acute inflammatory response, chemokine activity, cytokine-mediated signaling, negative regulation of immune processes, and TNF signaling (**Figure 4A**). Gene Set Enrichment Analysis (GSEA) further revealed upregulation of the NF-κB pathway (**Figure 4B**).

We then assessed sepsis-associated enhancement of canonical NF-κB signaling in colonic ILC3s. Flow cytometric analysis confirmed that while total NF-κB p65 protein levels remained unchanged in septic ILC3s (**Figure 4C**), the mean fluorescence intensity (MFI) of phosphorylated NF-κB p65 was significantly increased (**Figure 4D**). This was corroborated by immunofluorescence staining (**Figure 4E**) and Western blot analysis (**Supplementary Figure 4A**) on sorted ILC3 populations, which demonstrated enhanced phosphorylation and pronounced nuclear translocation of NF-κB p65 at 12 hours. Together, these results indicate that sepsis enhances NF-κB activation through increased p65 phosphorylation and nuclear translocation rather than through increased p65 expression. To determine the functional relevance of NF-κB enhancement, colonic ILC3s were sorted from septic mice at 12 hours post-LPS challenge and cultured ex vivo in the presence of the NF-κB nuclear translocation inhibitor 4-Methyl-N1- (3-phenylpropyl) benzene-1, 2-diamine (JSH-23). JSH-23 reduced IL-22 production in both control and septic ILC3s (**Figure 4F**), indicating that NF-κB activity contributes to IL-22 production under both basal and inflammatory conditions. In contrast, JSH-23 reduced GM-CSF production in septic ILC3s but had no significant effect on control ILC3s (**Figure 4G**), suggesting that the enhanced GM-CSF response during systemic inflammation is preferentially dependent on NF-κB activity. IL-17 production was not significantly altered by JSH-23 in either group (**Figure 4H**).

Intriguingly, stimulation of sorted WT ILC3s with TNF-α alone yielded a distinct cytokine profile. TNF-α induced GM-CSF production in a dose-dependent manner (**Supplementary Figure 3B**), but suppressed IL-22 secretion (**Supplementary Figure 3C**). This indicated that the coordinated production of both IL-22 and GM-CSF observed in septic ILC3s could not be recapitulated by TNF-α alone, suggesting a more complex, integrated signal from the septic microenvironment is required to drive their full functional activation.

### 3.5 Mitochondrial impairment promotes ROS accumulation in septic intestinal ILC3s

**Figure 5:**
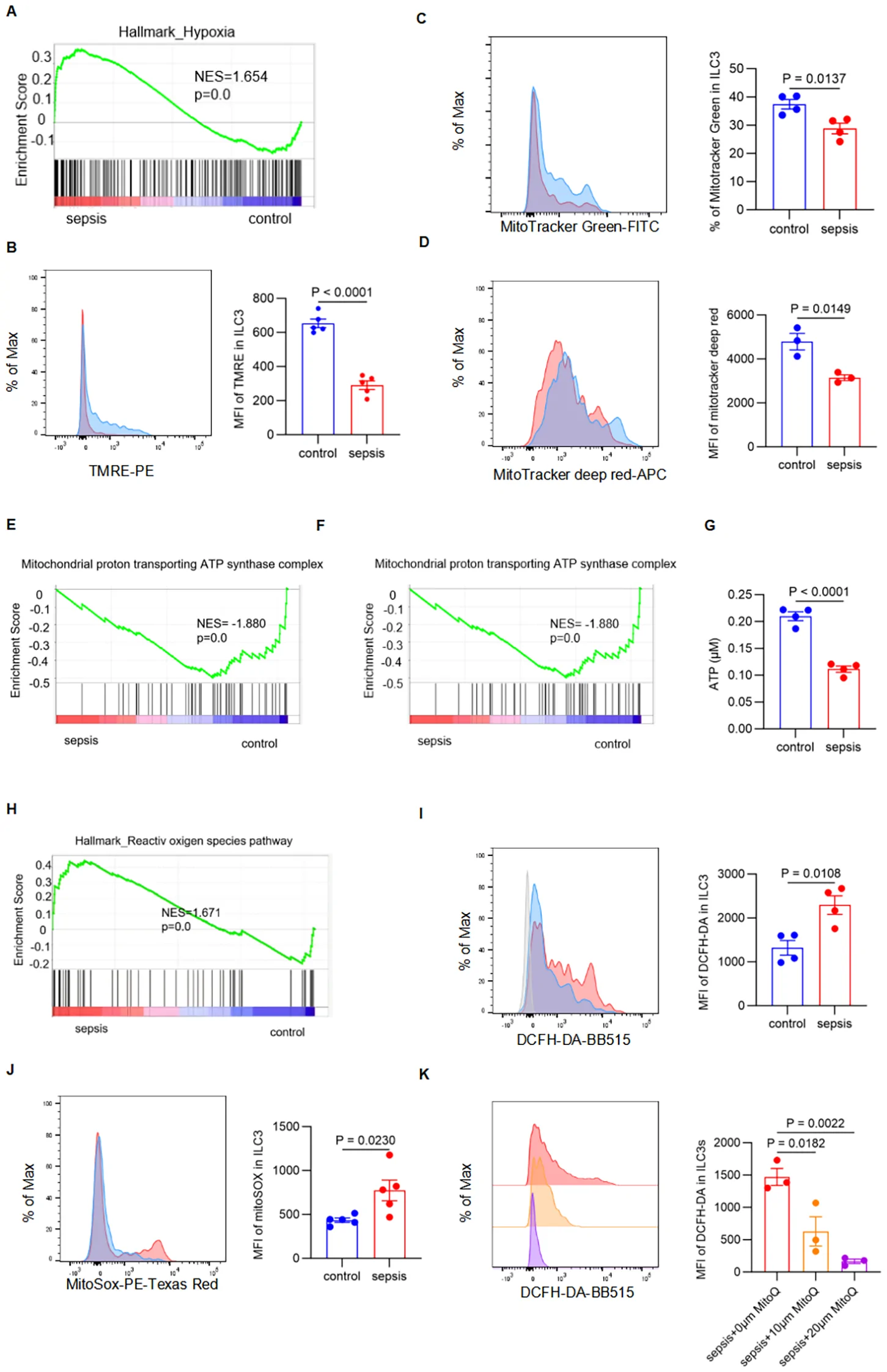
Mitochondrial impairment and ROS accumulation in septic intestinal ILC3s. Wild type mice were challenged with 20 mg/kg LPS i.p, while control mice received an equal volume of 1×PBS. Colon tissues were collected and analyzed at 12 hours. **(A)** GSEA was performed via the Broad Institute webtool. NES normalized enrichment score. **(B)** Representative flow cytometry plot and statistic of relative mean fluorescence intensity (MFI) of Tetramethylrhodamine ethyl ester (TMRE) in colonic ILC3s (n = 5). **(C)** Representative flow cytometry plot and statistic of relative MFI of MitoTracker™ green in colonic ILC3 (n = 4). **(D)** Representative flow cytometry plot and statistic of relative MFI of MitoTracker™ Deep Red in colonic ILC3 (n = 3). **(E, F)** GSEA was performed via the Broad Institute webtool. NES normalized enrichment score. **(G)** Intracellular ATP concentration was measured in sorted colonic ILC3s from septic and control mice using ATP assay kit (n = 4). **(H)** GSEA was performed via the Broad Institute webtool. NES normalized enrichment score. **(I)** Representative flow cytometry plot and statistic of relative MFI of 2’,7’-dichorofluorescin diacetate (DCFH-DA) in colonic ILC3 (n = 4). **(J)** Representative flow cytometry plot and statistic of relative MFI of MitoSox red mitochondrial superoxide indicator (MitoSOX™ Red) in colonic ILC3 (n = 5). Colonic ILC3s were sorted from sepsis mice induced by LPS i.p for 12 hours then treated with 0 μm,10 μm and 20 μm mitoquinone (MitoQ) which is a targeted mitochondrial antioxidant *in vitro* for 24 hours. **(K)** Representative flow cytometry plot and statistic of relative MFI of DCFH-DA in colonic ILC3 (n = 3). Data were pooled from two independent experiments **(A-K)**, shown as mean ± SEM. Statistical significance was determined by a two-tailed unpaired Student’s t test in **(B, C, D, G, I, J, K)**. Each symbol represents an individual mouse **(B, C, D, G, I, J, K)**. *p*-values are shown on the graphs, and *p*-values < 0.0001 are reported as such.

To further characterize the intracellular signaling mechanisms underlying ILC3 activation during sepsis, we performed Gene Set Enrichment Analysis (GSEA) on bulk RNA-seq data from colonic ILC3s. Gene sets related to hypoxia were significantly upregulated(**Figure 5A**). Athough GSEA revealed glycolysis-associated transcriptional remodeling (**Supplementary Figure 5A**), there is no significance on the uptake of the fluorescent glucose analog 2-NBDG (**Supplementary Figure 5B**). Concomitantly, both mitochondrial membrane potential, as assessed by tetramethylrhodamine ethyl ester (TMRE) (**Figure 5B**), and mitochondrial mass, as assessed by MitoTracker Green (**Figure 5C**) and Deep Red (**Figure 5D**), were reduced. Because the mitochondrial content was concurrently decreased, the reduction in TMRE signal may reflect changes in both mitochondrial abundance and membrane potential. Furthermore, GSEA identified downregulation of gene sets related to the mitochondrial proton transporting ATP synthase complex and ATP biosynthetic process (**Figures 5E, F**), which was confirmed by the detection of the decreased intracellular ATP levels in septic ILC3s (**Figure 5G**).

Mitochondria are recognized as the largest contributors to intracellular oxidant production (28). Consistent with the enrichment of reactive oxygen species-associated pathways (**Figure 5H**), both total intracellular ROS (**Figure 5I**) and mitochondrial ROS (**Figure 5J**) were increased in septic ILC3s. Treatment of septic ILC3s with the mitochondria-targeted antioxidant Mitoquenon (MitoQ) significantly reduced DCFH-DA fluorescence (**Figure 5K**). These results indicate that mitochondria make a substantial contribution to the increased cellular ROS burden but does not establish that all accumulated ROS are exclusively mitochondrial in origin.

*Nox4* expression was not significantly altered in septic ILC3s (**Supplementary Figures 5C, D**), although this finding does not exclude contributions from other ROS-generating enzymes. Increased MitoSOX fluorescence, together with the reduction in intracellular ROS following MitoQ treatment, supports a substantial mitochondrial contribution to the oxidative burden. Concurrent increases in *Sod2* expression (**Supplementary Figure 5E**) and NRF2 (**Supplementary Figure 5F**) abundance suggest induction of a compensatory antioxidant response that was insufficient to prevent ROS accumulation.

Collectively, these findings demonstrate that septic intestinal ILC3s undergo hypoxia-associated transcriptional remodeling accompanied by mitochondrial and bioenergetic impairment and increased oxidative stress.

### 3.6 ROS selectively regulates NF-κB activation and cytokine production in septic colonic ILC3s

**Figure 6:**
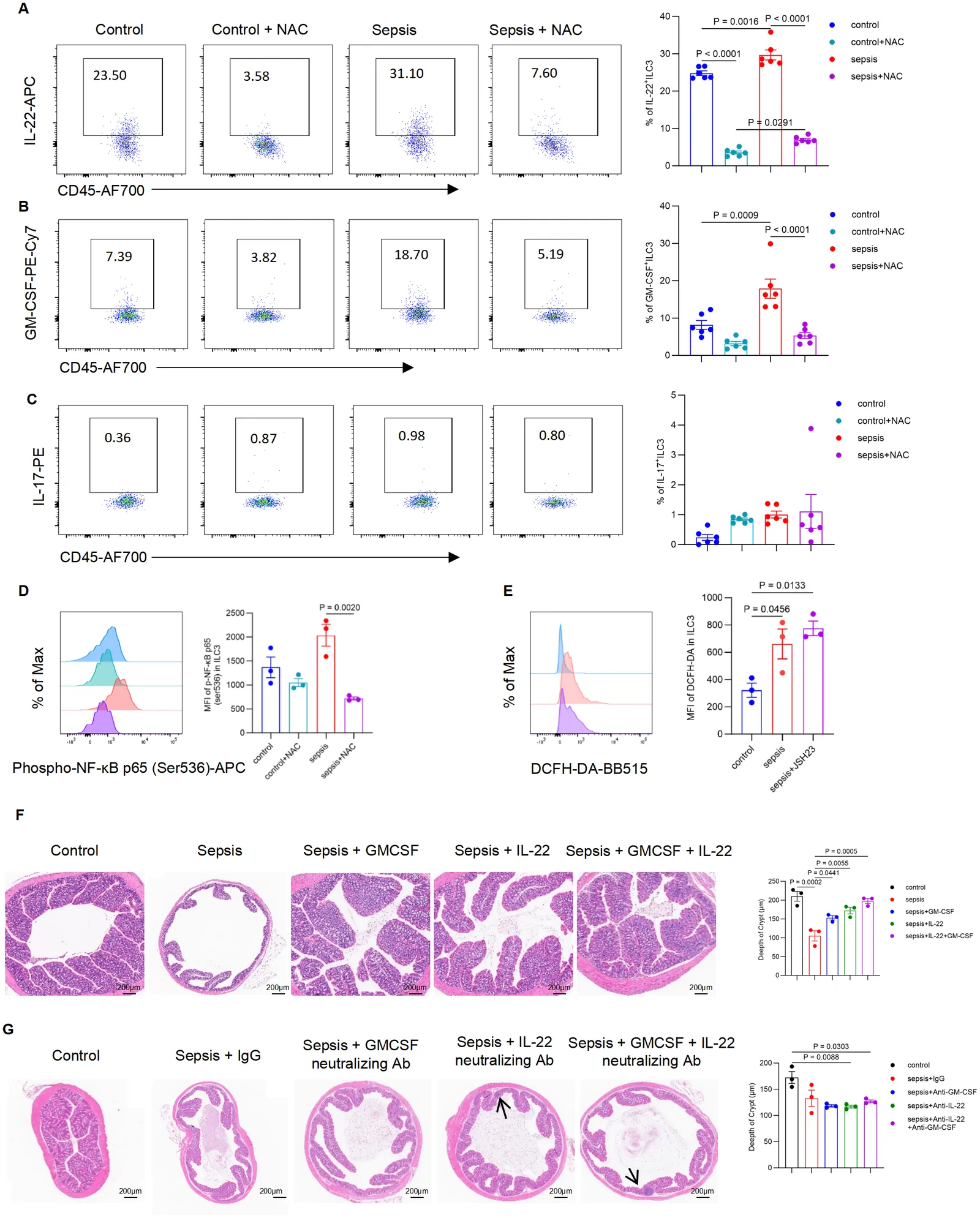
Mitochondrial ROS enhanced the production of IL-22 and GM-CSF in septic ILC3s by p-NF-κB p65. Colonic ILC3s were sorted from control and sepsis mice induced by PBS/LPS i.p for 12 hours then treated with 10 μM N- acetyl-L-cysteine (NAC) to erase ROS *in vitro* for 24 hours. Representative flow cytometry plot and statistic of frequency of IL-22 **(A)**, GM-CSF **(B)** and IL-17 **(C)** in sorting ILC3s after treating with NAC (n = 6/group). **(D)** Representative flow cytometry plot and statistic of relative MFI of p-NF-κB p65 in sorting ILC3s after treating with NAC (n = 3). Correspondingly, colonic ILC3s were sorted from control and sepsis mice induced by PBS/LPS i.p for 12 hours then treated with JSH-23 for 24 hours. **(E)** Representative flow cytometry plot and statistic of relative MFI of DCFH-DA in sorting ILC3s after treating with NAC (n = 3). **(F)** Representative image of H&E staining of colon sections from wild type (C57BL/6) septic mice supplemented with IL-22 (500 ng/per mice), GM-CSF (500 ng/per mice) or a combination of both cytokines and statistic of crypt depth. **(G)** Representative image of H&E staining of colon sections from wild type septic mice supplemented with IL-22 neutralizing antibody (50 μg/per mice). GM-CSF neutralizing antibody (50 μg/per mice) or a combination of both neutralizing antibodies and statistic of crypt depth. Data were pooled from two independent experiments **(A-G)**, shown as mean ± SEM. One-way ANOVA followed by Tukey’s multiple comparisons test in **(A-G)**. Each symbol represents an individual mouse **(A-G)**. *p*-values are shown on the graphs, and *p*-values < 0.0001 are reported as such.

To assess the functional consequences of ROS elevation, colonic ILC3s were sorted from mice at 0h and 12h post-LPS challenge and treated ex vivo with the ROS scavenger N-acetylcysteine (NAC). NAC reduced IL-22 secretion by both control and septic ILC3s (**Figure 6A**), indicating that mtROS contributes to sustaining IL-22 production under both basal and inflammatory conditions. NAC did not significantly affect GM-CSF secretion by control ILC3s but reduced GM-CSF production by septic ILC3s (**Figure 6B**), suggesting that the sepsis-associated GM-CSF response is preferentially dependent on mtROS. IL-17 production was not significantly altered by NAC in either group (**Figure 6C**). Together, these findings indicate that mtROS supports IL-22 production and the sepsis-associated GM-CSF response without broadly regulating ILC3 cytokine secretion.

Consistent with the increased p65 phosphorylation observed in septic ILC3s, NAC reduced phosphorylated p65 in septic ILC3s but had no significant effect in control cells(**Figure 6D**). Previous study has demonstrated that NF-κB can function as molecular redox switches by controlling activation/deactivation cycles in redox signaling (29). In a reciprocal experiment, inhibition of NF-κB nuclear translocation with JSH-23 did not affect intracellular ROS levels (**Figure 6E**), indicating that ROS accumulation precedes and promotes NF-κB activation rather than resulting from it. These findings support a model in which elevated ROS contributes to the enhanced NF-κB activation observed during sepsis.

Finally, given the altered production of IL-22 and GM-CSF observed in septic ILC3s, we examined whether exogenous administration of either cytokine could affect intestinal injury *in vivo*. Septic mice were treated with recombinant IL-22 or GM-CSF. Neither treatment affected serum inflammatory cytokines levels (**Supplementary Figure 6A**) or colonic inflammatory cytokines levels (**Supplementary Figure 6A**). However, both interventions attenuated colonic histopathological injury (**Figure 6F**). Treatment of septic wild-type mice with neutralizing antibodies against IL-22 or GM-CSF exacerbated colonic histopathological injury (**Figure 6G**). These findings demonstrate that both cytokines can reinforce mucosal antimicrobial defense and ameliorate intestinal barrier injury during systemic inflammation.

Collectively, these results support a context-dependent role for ROS in regulating NF-κB activation and cytokine production in septic ILC3s and demonstrate that exogenous IL-22 and GM-CSF can reinforce mucosal defense and mitigate intestinal barrier injury.

### 3.7 Impact of ROS-NF-κB signaling pathways on ILC3 survival

**Figure 7:**
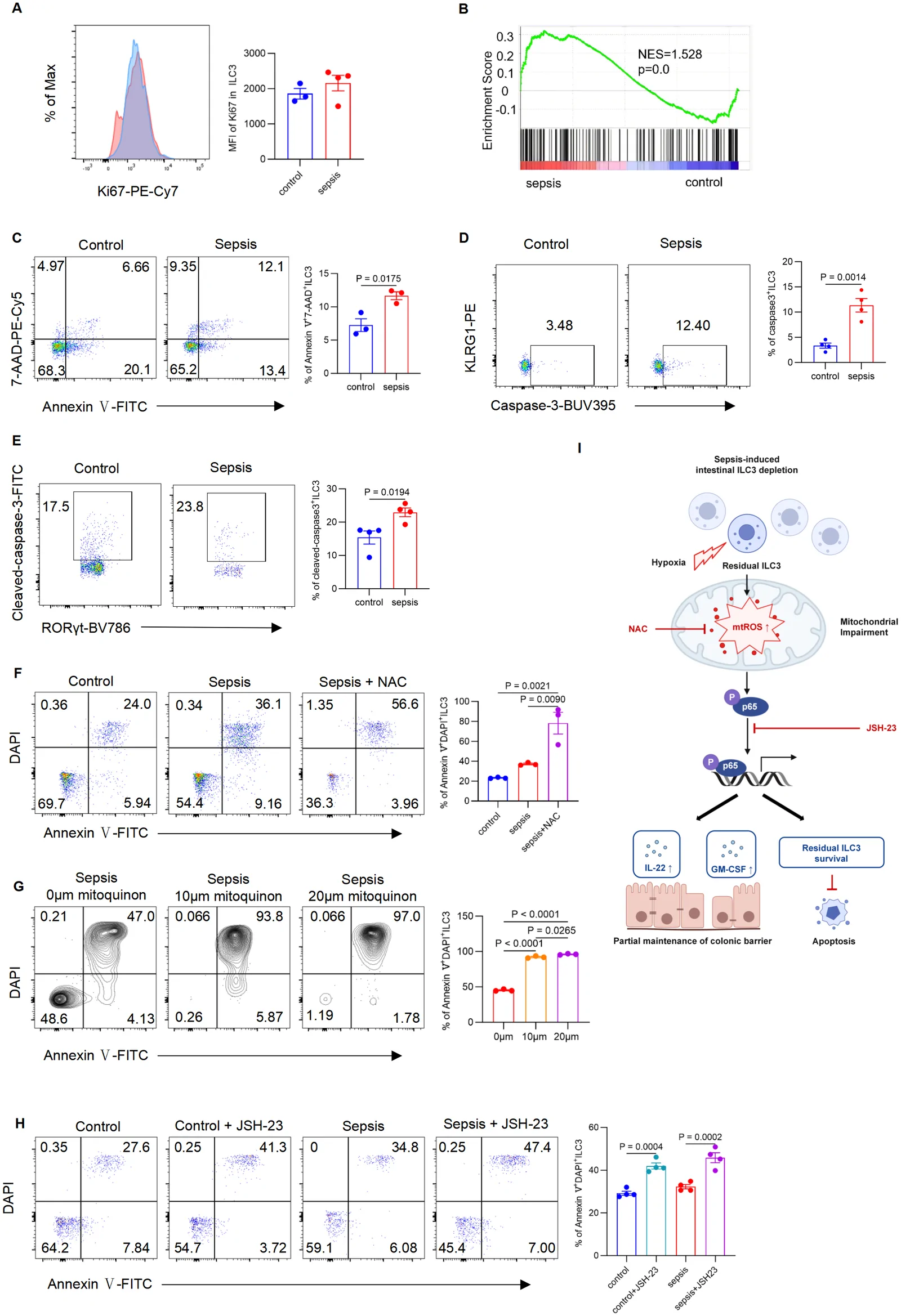
Inhibition of mtROS-NF-κB signaling promotes colonic ILC3s apoptosis during sepsis. Wild type mice were challenged with 20 mg/kg LPS i.p, while control mice received an equal volume of 1xPBS. Colon tissues were collected and analyzed at 12 hours. **(A)** Representative flow cytometry plot and statistic of relative MFI of Ki-67 in colonic ILC3 from control (n = 3) and sepsis (n = 4) mice. **(B)** GSEA was performed via the Broad Institute webtool. NES normalized enrichment score. Increased apoptosis of colonic ILC3s in septic mice. **(C)** Representative flow cytometry plot and statistic of frequency of Annexin V^+^7-AAD^+^ in colonic ILC3 (n =3). Representative flow cytometry plot and statistic of frequency of caspase-3 **(D)** and cleaved-caspase-3 **(E)** in colonic ILC3 (n =4). Colonic ILC3s were sorted from control and sepsis mice induced by PBS/LPS i.p for 12 hours then treated with three inhibitors for 24 hours to observe the apoptosis in colonic ILC3s, respectively. **(F)** Representative flow cytometry plot and statistic frequency of Annexin V^+^7-AAD^+^ in colonic ILC3s after treating with 0 and 10 mM NAC (n = 3). **(G)** Representative flow cytometry plot and statistic frequency of Annexin V^+^7-AAD^+^ in colonic ILC3s after treating with mitoquinon (n = 3). **(H)** Representative flow cytometry plot and statistic frequency of Annexin V^+^7-AAD^+^ in colonic ILC3s after treating with 10 mM JSH-23 (n = 3). **(I)** Proposed model of the mtROS-NF-κB axis in colonic ILC3s during sepsis. Data were pooled from two independent experiments **(A-H)**, shown as mean ± SEM. Statistical significance was determined by a two-tailed unpaired Student’s t test in **(A, C, D, E)**, one-way ANOVA followed by Tukey’s multiple comparisons test in **(F, G, H)**. Each symbol represents an individual mouse **(A, C-H)**. *p*-values are shown on the graphs, and *p*-values < 0.0001 are reported as such.

Given the reduction in ILC3 numbers, we first assessed their proliferative activity. Ki-67 expression did not differ between septic and control ILC3s (**Figure 7A**), suggesting that impaired proliferation was unlikely to account for the observed numerical decline. We therefore next examined cell-death-associated pathways. GSEA revealed enrichment of apoptosis-related pathways (**Figure 7B**), prompting us to determine whether ILC3s undergo apoptosis during sepsis. Flow cytometric analysis using Annexin V in combination with 7-AAD or DAPI showed a significant increase in the proportion of late apoptotic ILC3s at 12 hours relative to baseline (0 h) (**Figure 7C**). This finding was further supported by elevated levels of both total caspase-3 (**Figure 7D**) and cleaved caspase-3 (**Figure 7E**) in septic ILC3s. Numerous studies have demonstrated that mitochondrial dysfunction and the subsequent ROS elevation could lead to apoptosis (30–32). To determine whether the ROS-NF-κB axis participate in regulation of survival of septic ILC3, we performed complementary ex vivo inhibition experiments. Scavenging intracellular ROS with NAC significantly exacerbated apoptosis in septic ILC3s (**Figure 7F**). Similarly, treatment with MitoQ also increased the proportion of late apoptosis in septic ILC3s (**Figure 7G**). Furthermore, inhibition of NF-κB nuclear translocation with JSH-23 significantly increased late apoptotic cells in both steady-state and septic ILC3 (**Figure 7H**). Collectively, these data suggest that colonic ILC3s are subjected to substantial apoptotic stress during sepsis. Concurrent activation of the ROS-NF-κB signaling pathway appears to promote ILC3 survival, as inhibition of either upstream ROS or downstream NF-κB signaling removes this protective effect, leading to increased apoptosis.

Collectively, sepsis causes substantial depletion of intestinal ILC3s. The residual ILC3 population exhibits hypoxia-associated stress, mitochondrial impairment, and increased mtROS. Elevated mtROS contributes to NF-κB p65 phosphorylation and nuclear translocation, thereby supporting IL-22 and sepsis-associated GM-CSF production and promoting residual ILC3 survival by limiting apoptosis. These cytokine and survival responses partially maintain colonic barrier integrity but are insufficient to prevent ongoing ILC3 loss and intestinal injury. Pharmacological scavenging of ROS with NAC or JSH-23 disrupts this pathway(**Figure 7I**).

## 4 Discussion

In this study, we demonstrate that circulating ILCPs are significantly reduced in patients with sepsis. Using a complementary murine model, we show that despite numerical reduction, colonic ILC3s acquire enhanced effector capacity, characterized by increased production of IL-22 and GM-CSF. RORγt-deficient mice display impaired induction of colonic IL-22 and GM-CSF and aggravated intestinal barrier injury, whereas adoptive transfer of purified wild-type ILC3s partially restores cytokine production, mucosal antimicrobial defense, and barrier integrity. These findings provide functional evidence that ILC3s contribute directly to intestinal protection during sepsis, although they do not establish that this protection is mediated exclusively by ILC3-derived IL-22 or GM-CSF. Mechanistically, septic ILC3s undergo hypoxia-associated transcriptional remodeling accompanied by reduced mitochondrial mass and polarization-associated signals, decreased ATP availability, and accumulation of mitochondrial and intracellular ROS. Elevated ROS contributes to enhanced NF-κB p65 phosphorylation and selectively supports IL-22 and sepsis-associated GM-CSF production. At the same time, pharmacological scavenging of ROS or inhibition of NF-κB increases ILC3 apoptosis, suggesting that this pathway also provides compensatory survival signals to the residual ILC3 population. Thus, mitochondria-associated ROS-NF-κB signaling sustains both protective cytokine output and cell survival but is insufficient to fully prevent ongoing ILC3 loss and intestinal injury.

These findings reveal a dissociation between ILC3 abundance and effector activity during sepsis induced colonic injury: although ILC3 numbers declined, the residual population was enriched for IL-22- and GM-CSF-producing cells. The partial rescue achieved by adoptive transfer supports a direct protective contribution of ILC3s, while the supplementation and neutralization experiments establish barrier-protective roles for IL-22 and GM-CSF without demonstrating exclusive dependence on ILC3-derived cytokines. Nevertheless, this compensatory response was insufficient to offset ongoing ILC3 loss and progressive intestinal injury.

The role of NF-κB in regulating ILC3 effector programs appears context-dependent. It has been reported that NF-κB p65 binds the IL-22 promoter to promote human ILC3 proliferation and IL-22 production under the stimulation of IL-18 (33). In our study, septic ILC3s exhibited increased p65 phosphorylation and nuclear translocation without a corresponding change in total p65 abundance. Pharmacological inhibition of NF-κB reduced IL-22 production in both control and septic ILC3s, whereas GM-CSF was reduced only in septic ILC3s and IL-17 remained unchanged. These findings suggest that NF-κB supports IL-22 production under both basal and inflammatory conditions, while its contribution to GM-CSF becomes more prominent during sepsis. The cytokine-specific effects observed here differ from colitic settings in which NF-κB-dependent IL-17 or ROS-NF-κB-driven GM-CSF responses can promote intestinal inflammation (30, 31), further emphasizing that ILC3 effector programs are shaped by the inflammatory context.

The biological consequences of GM-CSF are similarly dependent on disease context, anatomical compartment, and timing. In anti-CD40-induced innate colitis, ILC3-derived GM-CSF promotes the recruitment and maintenance of inflammatory myeloid cells and contributes to tissue pathology (31). By contrast, exogenous GM-CSF in our acute systemic inflammation model ameliorated intestinal permeability and histopathological injury without broadly altering the measured systemic cytokine profile. These findings suggest that, in this setting, GM-CSF predominantly reinforces local mucosal defense rather than amplifying systemic inflammation. This interpretation is compatible with the immunostimulatory effects of GM-CSF reported in biomarker-selected patients with sepsis-associated immunosuppression, although clinical studies have not established a general mortality benefit from GM-CSF treatment in sepsis (32, 33).

Oxidative stress exhibits a dual nature. Excessive oxidative burden leads to cellular injury, while owing to intrinsic antioxidant defenses, modest and transient elevations in ROS are likely to act as signaling intermediates that coordinate adaptive physiological responses (34–36). Additionally, NF-κB, a canonical redox-responsive transcription factor, promotes cell survival in part through transcriptional activation of its downstream target B cell lymphoma 2 (BCL-2), a key anti-apoptotic protein (37). The present findings primarily define the role of mtROS-NF-κB signaling under sepsis-associated hypoxia stress and do not establish that sustained activation of this pathway is required for ILC3 function under steady-state conditions. Basal mitochondrial ROS may contribute to physiological intracellular signaling. However, excessive mtROS accumulation during sepsis appears to activate an adaptive program that promotes NF-κB-dependent IL-22 and GM-CSF production and supports the survival of the remaining ILC3 population. Nevertheless, this response is insufficient to overcome the concurrent mitochondrial dysfunction, bioenergetic stress, and apoptotic pressure induced by sepsis. Because NAC, MitoQ, and JSH-23 are pharmacological agents, potential compound-specific or viability-related effects cannot be excluded, and the proposed survival mechanism requires confirmation using ILC3-specific genetic approaches.

Several limitations should be acknowledged. First, although our data support a role for mitochondria-associated ROS-NF-κB signaling in septic ILC3 activation, the upstream sensors and metabolic pathways involved remain undefined. Low ILC3 yields precluded metabolomic profiling, and future low-input metabolomic and genetic approaches will be required for deeper mechanistic analysis. Second, although enhanced apoptosis contributed to ILC3 loss, its proximal triggers were not identified. Mitochondrial damage, mtDNA release, and defective mitophagy are plausible mechanisms that warrant further investigation using ultrastructural, molecular, and functional assays. Third, although adoptive transfer of purified wild-type ILC3s improved intestinal permeability, histopathological injury, and antimicrobial peptide expression in RORγt-deficient mice, survival following ILC3 reconstitution was not assessed. Therefore, the present findings support a barrier-protective effect of ILC3s but do not establish an improvement in overall disease outcome or mortality. Future studies using adequately powered cohorts will be required to determine whether ILC3 reconstitution confers a survival benefit.

## 5 Conclusion

Collectively, our findings identify a compensatory mitochondria-associated ROS-NF-κB program in septic intestinal ILC3s. This program selectively sustains IL-22 and GM-CSF responses and supports survival of the residual ILC3 population, thereby reinforcing mucosal antimicrobial defense and intestinal barrier protection. However, the magnitude of this activation is insufficient to prevent continued ILC3 loss and progressive intestinal injury during sepsis.

## Data Availability

The datasets presented in this study can be found in online repositories. The names of the repository/repositories and accession number(s) can be found below: https://www.ncbi.nlm.nih.gov/geo/, GSE322583.
