# Supplementary material for: mtROS-NF-κB signaling supports ILC3 function and survival during sepsis-induced intestinal injury
Source: Front Immunol. 2026 Sep 9;17:1843175. doi: 10.3389/fimmu.2026.1843175 (PMC13597377; doi:10.3389/fimmu.2026.1843175)
Supplement: Supplementary file 1 [file DataSheet1.docx]

Supplementary figure 1. The comparison between septic patients with intestinal perforation and non-intestinal perforation.

(A and B) Severity of sepsis patient. SOFA scores (A) and APACHEⅡ(B) among septic patients admission in ICU 0 day (n = 22)、3 days (n = 13) and 7 days (n = 8). (C) Severity comparison between septic patients with intestinal perforation and non-intestinal perforation presented as SOFA scores. (n = 14 for non-intestinal perforation and n=8 for intestinal perforation). (D) The cell number of white blood cells (WBC), neutrophils, lymphocytes, and monocytes in peripheral blood between septic patients without intestinal perforation (n = 13) and with intestinal perforation (n = 7). (E) The frequency and number of live CD45^+^ cells between septic patients without intestinal perforation (n = 11) and with intestinal perforation (n = 5). (F)The frequency and number of helper-like ILCs in CD45^+^ cells between septic patients without intestinal perforation (n = 11) and with intestinal perforation (n = 5). (G) The frequency and number of ILC1-like cells in ILCs between septic patients without intestinal perforation (n = 11) and with intestinal perforation (n = 5). (H) The frequency and number of ILC2s in ILCs between septic patients without intestinal perforation (n = 11) and with intestinal perforation (n = 5). (I) The frequency and number of ILCPs in ILCs between septic patients without intestinal perforation (n = 11) and with intestinal perforation (n = 5). Data are presented as mean ± SEM. Each symbol represents an individual sample. For statistical analysis, the following tests were used. One-way ANOVA followed by Tukey’s multiple comparisons test in (A, B, D) and two-tailed unpaired Student’s t-test (C, E, F, G, H). *p*-values are shown on the graphs, and *p*-values < 0.0001 are reported as such.

Supplementary figure 2. Intestinal injury occurs in LPS-induced sepsis mice.

Wild type mice were challenged by LPS (20mg/kg) intraperitoneal injection (i.p) to induce sepsis model, while control mice received an equal volume of 1xPBS. Serum and colon tissues were collected and analyzed at 0 h, 12 h and 24h. (A) Survival was monitored every 12 hours for 72 hours. (n = 18/group, Log-Rank test). (B) Dynamic changes in plasma inflammatory cytokine profiles following sepsis induction. Plasma concentrations of pro-inflammatory cytokine (INF-γ, TNF-α, MCP-1, IL-1β, IL-6) and the anti-inflammatory cytokine IL-10 were measured using a multiplex immunoassay at 0, 12, and 24 hours after LPS injection (n = 4 per group per time point). (C) Representative image and statistic of crypt depth of H&E staining of colon sections from sepsis mice at 0 h,12 h, and 24 h (n=4/group). Arrows indicate inflammatory cell infiltration. The scale bar is 200 μm. Each symbol represents an individual mouse. (D) Representative Alcian Blue-Periodic Acid-Schiff (AB-PAS) staining and statistic of colon sections and histological quantification of AB-PAS+ (positive) area by Image J (n=4/group). Scale bars, 200 μm. (E) Intestinal permeability of sepsis mice at 0h, 12h and 24h detected by dextran assay (n = 4/group) (F) Dynamic changes in colon inflammatory cytokine profiles following sepsis induction. Colon tissue concentrations of pro-inflammatory cytokines (INF-γ, TNF-α, MCP-1, IL-1β, IL-6) and the anti-inflammatory cytokine IL-10 were measured using a multiplex immunoassay at 0,12 and 24 hours after LPS injection (n = 4 per group per time point). (G) Expression of *Reg3a*, *Reg3b* and *Reg3g* in large intestine at 0,12, and 24 hours after LPS injection (n = 4 per group per time point). Data were pooled from two independent experiments (A-G), shown as mean ± SEM. Statistical significance was determined by Kaplan-Meier survival analysis (A), one-way ANOVA followed by Tukey’s multiple comparisons test in (B, E, F, G). Each symbol represents an individual mouse (B, E, F, G). *p*-values are shown on the graphs, and *p*-values < 0.0001 are reported as such.

Supplementary figure 3. Identification of ILC3 deficiency and the influence of IL-22 and CM-CSF on inflammation in sepsis mice.

(A) Representative flow cytometry plots of ILC3s and ILC2s gate on Lin^-^CD45^+^ in *Rorc^+/+^* and *Rorc*^gfp/gfp^ mice. Wild-type mice were challenged with LPS i.p to induce sepsis. Colon tissues were collected at 12 hours, and ILC3s were sorted for western blot analysis. (B) Concentration of serum pro-inflammatory cytokines included INF-γ, IL-1β and anti-inflammatory cytokine IL-10 in *Rorc^+/+^* and *Rorc^gfp/gfp^* mice in steady state and sepsis model. Data were shown as mean ± SEM. Statistical significance was determined by two-way ANOVA followed by Tukey’s multiple comparisons test in (B). Each symbol represents an individual mouse (B). *p*-values are shown on the graphs, and *p*-values < 0.0001 are reported as such.

Supplementary figure 4. NF-κB signaling participates in regulating the secretion of cytokines in colonic ILC3 during sepsis.

(A) Wild-type mice were challenged with LPS i.p to induce sepsis. Colon tissues were collected at 12 hours, and ILC3s were sorted for western blot analysis. The experiments were repeated two times with similar results, and the representative immunoblot images and statistic were shown. Phosphorylated NF- κB p65 (ser 536) and total NF- κB p65 expression by western blot. (B ) Colonic ILC3s sorted from wild-type mice were treated with different concentrations (0 ng/mL, 1 ng/mL, 10 ng/mL, 50 ng/mL, 100 ng/mL) TNF-α in the presence of IL-7 (10 ng/ml) for 24 hours to observe the production of GM-CSF and IL-22 by flow cytometry, the ILC3s sorted from the colon of LPS-induced mice and cultured for 24 hours in medium without TNF-α as a control (n = 3) . Data were pooled from two independent experiments (A-B), shown as mean ± SEM. Statistical significance was determined by one-way ANOVA followed by Tukey’s multiple comparisons test in (A, B). Each symbol represents an individual mouse (A, B). *p*-values are shown on the graphs, and *p*-values < 0.0001 are reported as such.

Supplementary figure 5. Differential expression of oxidative and antioxidant molecules in RNA sequencing colonic ILC3s during sepsis.

Wild-type mice were challenged with LPS i.p to induce sepsis. Colon tissues were collected at 12 hours, and ILC3s were sorted for RNA sequencing. (A) GSEA was performed via the Broad Institute webtool. NES normalized enrichment score. (B) Representative flow cytometry plot and statistic of relative mean fluorescence intensity (MFI) of 2-(N-[7-nitrobenz-2-oxa-1,3-diazol-4-yl] amino) −2-deoxyglucose (2-NBDG) in colonic ILC3 (n = 5). (C) Heatmap of antioxidant and NADPH oxidase. (D) FPKM value of *Nox4* encoded NADPH oxidase. (E) FPKM value of superoxide dismutase (*Sod*). (D) Representative flow cytometry plot and statistic of nuclear respiratory factor 2 (NRF2) . Data were shown as mean ± SEM. Statistical significance was determined by a two-tailed unpaired Student’s t test in (B, C, D, E). Each symbol represents an individual mouse. *p*-values are shown on the graphs, and *p*-values < 0.0001 are reported as such.

Supplementary figure 6. Treantments with IL-22 and GM-CSF don’t have influence on serum and colonic inflammatory cytokines.

(A) Concentration of serum pro-inflammatory cytokines included INF-γ, TNF-α, MCP-1, IL-1β, IL-6 and anti-inflammatory cytokine IL-10 in wild type septic mice supplemented with IL-22 (500 ng/per mice), GM-CSF (500 ng/per mice) or a combination of both cytokines by multiplex immunoassay. (B) Concentration of colonic tissue pro-inflammatory cytokines included INF-γ, TNF-α, MCP-1, IL-1β, IL-6 and anti-inflammatory cytokine IL-10 in wild type septic mice supplemented with IL-22 (500 ng/per mice), GM-CSF (500 ng/per mice) or a combination of both cytokines by multiplex immunoassay. Data were shown as mean ± SEM. Statistical significance was determined by one-way ANOVA followed by Tukey’s multiple comparisons test in (A, B). Each symbol represents an individual mouse (A, B). *p*-values are shown on the graphs, and *p*-values < 0.0001 are reported as such.

Supplementary Table 1. The characteristics of sepsis patients at the admission of ICU.

|  | Healthy control (n=9) | Sepsis patients (n=22) |
| --- | --- | --- |
| Gender（male,%) | 5(50.00) | 13(59.09) |
| Age | 64±16 | 61±15 |
| **Comorbidity** |  |  |
| Hypertension | ND | 9(40.91) |
| Diabetes | ND | 7(31.82) |
| Chronic kidney disease | ND | 1(4.54) |
| Chronic obstructive pulmonary disease | ND | 2(9.09) |
| Chronic cardiac insufficiency | ND | 2(9.09) |
| Chronic hepatic insufficiency | ND | 0(0.0) |
| Connective tissue disease | ND | 1(4.54) |
| Tumor | ND | 6(27.27) |
| others | ND | 5(22.73) |
| Corticosteroid use | ND | 2(9.09) |
| Acute kidney injury | ND | 12(54.55) |
| **Infection site** |  |  |
| lung | ND | 12(54.55) |
| abdomen | ND | 9(40.90) |
| Urinary tract | ND | 2(9.09) |
| Skin and soft tissue | ND | 1(4.54) |
| Bloodstream | ND | 1(4.54) |
| Brain | ND | 1(4.54) |
| Survival | ND | 15(68.18) |

Supplementary Table 2. Antibody used for flow cytometry

| Antibody | Fluorescence channel | RRID | Company |
| --- | --- | --- | --- |
| Anti-mouse CD45 | AF700 | AB_493730 | Biolegend |
| Anti-mouse/human(CD45R) B220 | FITC | AB_312990 | Biolegend |
| Anti-mouse CD3e | FITC | AB_312670 | Biolegend |
| Anti-mouse/human CD11b | FITC | AB_312788 | Biolegend |
| Anti-mouse CD11c | FITC | AB_313774 | Biolegend |
| Anti-mouse GR-1 | FITC | AB_313370 | Biolegend |
| Anti-mouse/human RORγt | BV786 | AB_2738916 | BD Bioscience |
| Anti-mouse T-Bet | PE | AB_11218985 | Biolegend |
| Anti-mouse GATA3 | BB700 | AB_2563218 | Biolegend |
| Anti-mouse Nkp46 | BV510 | AB_2738217 | BD Bioscience |
| Anti-mouse CCR6 | BV421 | AB_10898320 | Biolegend |
| Anti-mouse IL-22 | APC | AB_2563355 | Biolegend |
| Anti-mouse IL-17A | PE | AB_315463 | Biolegend |
| Anti-mouse IFN-γ | BV605 | AB_2561438 | Biolegend |
| Anti-mouse GM-CSF | PE-CY7 | AB_2721681 | Biolegend |
| Anti-human CD3 | FITC | AB_314059 | Biolegend |
| Anti-human TCRαβ | FITC | AB_314639 | Biolegend |
| Anti-human TCRγδ | FITC | AB_1575111 | Biolegend |
| Anti-human CD19 | FITC | AB_314235 | Biolegend |
| Anti-human CD20 | FITC | AB_314251 | Biolegend |
| Anti-human CD1a | FITC | AB_1724015 | eBioscience |
| Anti-human CD14 | FITC | AB_314185 | Biolegend |
| Anti-human CD16 | FITC | AB_10805747 | eBioscience |
| Anti-human CD123 | FITC | AB_755989 | Biolegend |
| Anti-human CD303 | FITC | AB_2561363 | Biolegend |
| Anti-human FcεR1a | FITC | AB_10732835 | eBioscience |
| Anti-human CD34 | FITC | AB_1731923 | Biolegend |
| Anti-human CD94 | FITC | AB_314534 | Biolegend |
| Anti-human CD45 | AF700 | AB_2566373 | Biolegend |
| Anti-human CD127 | PE | AB_10853334 | eBioscience |
| Anti-human CD117 | BV421 | AB_10896056 | Biolegend |
| Anti-human CRTH2 | PE-CF594 | AB_2738244 | BD Bioscience |
| Anti-human Nkp44 | BB700 | AB_2616751 | Biolegend |
| Anti-human CD161 | BV605 | AB_11142679 | Biolegend |
| Anti mouse CD3 | PE-CF594 | AB_11153307 | BD Bioscience |
| Anti mouse B220 | PE-CF594 | AB_11151901 | BD Bioscience |
| Anti mouse GR-1 | PE-CF594 | AB_2737737 | BD Bioscience |
| Anti mouse CD11b | PE-CF594 | AB_11154422 | BD Bioscience |
| Anti mouse CD11c | PE-CF594 | AB_2737617 | BD Bioscience |
| Anti mouse CD4 | PE-CF594 | AB_493646 | Biolegend |
| Anti-mouse CD3e | APC-CY7 | AB_1877171 | Biolegend |
| Anti-mouse (CD45R) B220 | APC-CY7 | AB_313006 | Biolegend |
| Anti-mouse CD11b | APC-CY7 | AB_830641 | Biolegend |
| Anti-mouse CD11c | APC-CY7 | AB_830646 | Biolegend |
| Anti-mouse GR-1 | APC-CY7 | AB_2137486 | Biolegend |
| Anti-mouse CD90 | BV605 | AB_2632889 | Biolegend |
| Anti-mouse CD90 | PE-CY7 | AB_2563357 | Biolegend |
| Anti-mouse/human KLRG-1 | PE | AB_10574005 | Biolegend |
| Anti-mouse KLRG-1 | BV605 | AB_2563357 | Biolegend |
| Anti-mouse NK1.1 | BB700 | AB_2132706 | Biolegend |
| Anti-mouse Ki67 | PE-CY7 | AB_2910305 | BD Bioscience |
| Active Caspase-3 apop kit | BUV395 | AB_3685569 | BD Bioscience |
| Live dead fixable dead cell kit | BV-510 | AB_2869572 | BD Bioscience |
| Anti-mouse CD45 | AF700 | AB_493730 | Biolegend |

Supplementary Table 3. Primers used in this study

| Gene | Forward | Reverse |
| --- | --- | --- |
| *Actin* | GGCTGTATTCCCCTCCATCG | CCAGTTGGTAACAATGCCATGT |
| *Reg3a* | TCACCTGGTCCTCAACAGTATT | GGAGCGATAAGCCTTGTAACC |
| *Reg3b* | ACTCCCTGAAGAATATACCCTCC | CGCTATTGAGCACAGATACGAG |
| *Reg3g* | ATGCTTCCCCGTATAACCATCA | GGCCATATCTGCATCATACCAG |
